# The g.-165 T>C Rather than Methylation Is Associated with Semen Motility in Chinese Holstein Bulls by Regulating the Transcriptional Activity of the *HIBADH* Gene

**DOI:** 10.1371/journal.pone.0127670

**Published:** 2015-07-02

**Authors:** Shuai Zhang, Yan Zhang, Chunhong Yang, Zhihua Ju, Xiuge Wang, Qiang Jiang, Yan Sun, Jinming Huang, Jifeng Zhong, Changfa Wang

**Affiliations:** 1 Dairy Cattle Research Center, Shandong Academy of Agricultural Science, Jinan, 250131, PR China; 2 College of Life Science, Shandong Normal University, Jinan, 250014, PR China; Clermont-Ferrand Univ., FRANCE

## Abstract

The 3-hydroxyisobutyrate dehydrogenase (HIBADH) is regarded as a human sperm-motility marker. However, the molecular mechanisms involved in the regulation of expression of the *HIBADH* gene in bulls remain largely unknown. *HIBADH* was detected in the testis, epididymis, and sperm via reverse transcription polymerase chain reaction and Western blot analysis. It is also expressed in the seminiferous epithelium, spermatids, and the entire epididymis, as detected by immunohistochemistry. Furthermore, HIBADH was expressed in the neck-piece and mid-piece of bull spermatids, as shown in the immunofluorescence assay. Using serially truncated bovine *HIBADH* promoters and luciferase constructs, we discovered an 878 bp (-703 bp to +175 bp) fragment that constitutes the core promoter region. One SNP g.-165 T>C of *HIBADH* was identified and genotyped in 307 Chinese Holstein bulls. Correlation analysis revealed that bulls with the TT genotype had higher initial sperm motility than those with the CC genotype (*P < 0*.*05*). Furthermore, the T- or C-containing loci (designated as pGL3-T and pGL3-C) were transiently transfected into MLTC-1 to test the effect of SNP on HIBADH expression. The luciferase reporter assay showed that the pGL3-T genotype exhibited 58% higher transcriptional activity than the pGL3-C genotype (*P < 0*.*05*). The bisulfite sequencing analysis revealed that the methylation pattern of the core promoter presented hypomethylation in the ejaculated semen in high-motility and low-motility bulls. The results demonstrated for the first time that the g.-165 T>C rather than methylation in the 5'-flanking region could affect the bovine sperm motility through the regulation of *HIBADH* gene transcriptional activity.

## Introduction

Artificial insemination (AI), an assisted reproductive technology protocol, is a valuable technique that is implemented in bovine reproduction [1]. AI greatly improves the breeding efficiency in dairy cattle herds. However, the conception rate with AI depends on the quantity and quality of semen. The parameters for the quantity and quality of semen, including the ejaculate volume, sperm density, initial sperm motility, post-thaw cryopreserved sperm motility, and sperm deformity rate, are quantitative traits that are affected by heredity, environment, management, and physiological status [2]. The sperm concentration and motility in traditional semen analysis measures are poor predictors of fertility and demonstrate remarkable intra- and inter-individual variability [3]. Because of these limitations, sperm molecular biomarkers that may better and more stably reflect sperm function were developed. In the past, many similar works have been performed on goats [4] and boars [5–7]. However, only a few studies focused on the candidate marker genes in bulls [8–17].

The 3-hydroxyisobutyrate dehydrogenase (HIBADH), a metabolism-related protein, is a critical enzyme that generates glucose by metabolizing amino acids in the gluconeogenesis pathway [18]. HIBADH participates in valine, leucine, and isoleucine degradation and is a central metabolic enzyme in the valine catabolic pathway [19–22]. The 3-hydroxy-2-methylpropanoate is used with NAD^+^ as the substrate, and the three products are 2-methyl-3-oxopropanoate, NADH, and H^+^. HIBADH is primarily expressed in the mitochondria of various tissues and cells [20, 23]. In neural cells, HIBADH may use the valine that is imported into the brain for energy generation [24]. Previous reports suggested that HIBADH expression is elevated in the neck-piece and mid-piece of the elongating, elongated, and mature sperms that contain the mitochondria during human spermiogenesis [25]. Several studies showed that HIBADH is a motility-related protein, as analyzed by proteomic approach [26–27] and has genome-wide association in Holstein-Friesian bulls [28]. These data indicated that HIBADH may be involved in maintaining sperm motility [25] and may function as a sperm motility marker. However, its detailed expression patterns in different bull organs and semen have not been fully characterized.

Functional single nucleotide polymorphisms (SNPs) are the most common forms of genetic variation with far-reaching influence in many aspects of the mammalian genome, such as protein coding and expression regulation. The 5′-flanking region of the gene, particularly the minimal promoter, is the key transcriptional regulatory region in gene expression [29–30]. Furthermore, as a stable epigenetic modification, DNA methylation is an important regulator in a number of biological processes, including testicular development and spermatogenesis [31]. Genetically, DNA methylation usually occurs in sequences with a CpG island, and such sequences are located in the promoter regions of genes in differentially methylated regions or in imprinting control regions [32–33]. Correct DNA methylation plays an important role in the regulation of spermatogenesis because hypermethylation is correlated with abnormal sperm parameters, idiopathic male factor infertility, and pregnancy failure [34].

Based on the abovementioned description, we suggested that bovine *HIBADH* affects sperm quality traits. To confirm our hypotheses, the following studies were performed. (i) We determined the expression and localization of the *HIBADH* gene in Chinese Holstein bulls using reverse transcription polymerase chain reaction (RT-PCR), Western blot analysis, immunohistochemistry (IHC), and immunofluorescence assay (IFA). (ii) We investigated potentially functional genetic variants in the 5′-flanking region of *HIBADH* and their relationship with semen quality traits in Chinese Holstein bulls. (iii) We determined the core promoter region and the effect of the genetic variants on the transcription of the *HIBADH* gene. (iv) We identified the promoter methylation pattern of the *HIBADH* 5’-flanking region in bull sperms with high or low motility.

## Materials and Methods

### Ethics statement

All experiments were performed according to the Regulations for the Administration of Affairs Concerning Experimental Animals published by the Ministry of Science and Technology, China in 2004. The study involving bull semen and tissue samples was approved by the Animal Care and Use Committee in Shandong Academy of Agricultural Sciences, Shandong, P. R. China. Collection of semen and tissue samples was permitted by the animal owners, and the samples were collected by the workers of the companies.

### Semen and tissue samples

Semen samples were collected from a total of 307 Chinese Holstein bulls from three bull stations (65 bulls from the Shandong OX Bio-Technology Co., Ltd.; 92 bulls from the Beijing Dairy Center; and 150 bulls from the Shanghai Bright Dairy and Food Co., Ltd.). The semen quality traits included ejaculate volume, sperm concentration, initial sperm motility, post-thaw cryopreserved sperm motility, and sperm deformity rate, as described by Liu *et al*. (2011)[11]. For each bull, repeated measurements of sperm quality traits were performed from 2005 to 2012. The ejaculate volume was measured in a semen-collecting vial, and the number of sperm cells was counted. The sperm concentration was calculated using a sperm densitometer (Accucell; IMV Biotechnology, L’Aigle, France). The motilities of the fresh and post-thaw cryopreserved sperms were viewed on a TV monitor, which was connected to a camera mounted onto a phase-contrast microscope (Olympus-BX40; Optical Co., Ltd.) at 400× magnification. The percentage of sperm deformities was determined at 400× and 1,000× magnification with Giemsa stain [35]. To minimize variation, the quality trait assessment of all the semen samples in each semen collection station was performed by a well-trained technician. The semen samples were used for RT-PCR, Western blot, and immunofluorescence analyses. The means and standard errors of the sperm quality traits of the 307 Chinese Holstein bulls are shown in Table 1. The 307 genotyped bulls were sons of 187 sires (each sire had 1 to 5 sons; on average, 1.64 sons). These materials may be applied for SNP screening, genotyping, and association analyses.

**Table 1:** Means and standard errors (SE) of sperm quality traits in 307 Chinese Holstein bulls.

Traits	Mean ± SE
Ejaculate volume (mL)	6.21 ± 0.11
Initial sperm motility (%)	74.04 ± 0.24
Sperm density (×10^8^/mL)	11.02 ± 0.24
Frozen/thawed sperm motility (%)	42.03 ± 0.68
Deformity rate (%)	14.67 ± 0.29

Tissue samples, including those from heart, liver, spleen, lung, kidney, testis, and epididymis, were collected from three randomly selected euthanized adult Chinese Holstein bulls (3 years old) from the farms of the Dairy Cattle Research Center, Shandong Academy of Agricultural Sciences. The tissue samples were approved by the Animal Care and Use Committee in Shandong Academy of Agricultural Sciences. The tissue samples were collected, frozen in liquid nitrogen, and immediately stored at -80°C. These samples were used for DNA extraction, Western blot analysis, and IHC synthesis to determine the expression pattern of the *HIBADH* gene. In addition, fresh semen samples were obtained from six adult Holstein bulls (7.06 years old) from Shandong OX Bio-Technology Co., Ltd. Six bulls were assigned to two groups based on sperm motility. The two groups included the high-motility group (sperm motility, ≥75%; sperm concentration, ≥6 × 10^8^/mL; and abnormal sperm percentage, ≤15%) and the low-motility group (50% ≤ sperm motility ≤ 60%). The fresh semen samples were maintained at 37°C and immediately returned to the laboratory for the investigation on the core promoter methylation pattern of *HIBADH*. Fresh testicular and epididymal samples from two adult bulls were fixed in 4% paraformaldehyde for 48 h at room temperature, embedded in paraffin, and cut into 7 μm sections for bovine HIBADH protein localization. The murine Leydig tumor cells (MLTC-1) were obtained from the Cell Culture Collection of the Chinese Academy of Sciences, Shanghai, China.

### Sperm DNA extraction

The genomic DNA was extracted from the sperm samples of bulls using the standard protocol that was described by Gao et al. (2014)[15]. Lysis solution (80 mL) containing 12.5 mM ethylenediaminetetraacetic acid (EDTA; pH 8.0), 12.5 mM Tris-HCl (pH 8.0), 0.4 M dithiothreitol, 0.4 M NaCl, and 12.5% SDS with 20% SDS and proteinase K (20 mg/mL) was added to the sperm pellet. The mixed liquid was digested at 56°C for approximately 10 h. The genomic DNA was separated using saturated salt water and precipitated using precooled 100% ethanol. The DNA was washed twice in 75% ethanol and dried for several minutes. Finally, the DNA was dissolved in Tris-EDTA buffer containing 10 mM Tris-HCl (pH 7.5) and 1 mM EDTA (pH 8.0). All DNA samples were stored at -20°C for subsequent analyses.

### Isolation of RNA and cDNA synthesis

Total RNA was isolated from semen by following a previously published protocol [15]. The somatic cells in semen were lysed in 1 mL cell lysis solution (0.1% SDS and 0.5% Triton X-100) and subsequently washed in 1 mL rinsing solution (60% Tris-OH, 8.6% sucrose). The sperm cells were lysed in 1 mL Trizol (Invitrogen), and the proteins were removed using 200 μL of chloroform. The total RNA was precipitated using an equal volume of isopropanol, and the RNA pellet was washed with 75% ethanol. Finally, the total RNA was dissolved in 20 μL diethylpyrocarbonate-treated water. Complementary DNA was synthesized using a transcript first-strand cDNA synthesis kit (TaKaRa, Dalian, China). The total RNA was extracted from tissues, and reverse transcription to cDNA was performed according to a previously published protocol [17].

### Western blot, IHC, and IFA

Western blot analysis was performed according to standard protocols [14]. The tissue samples from the adult bulls were homogenized using radio-immunoprecipitation assay lysis buffer (Beyotime, China). After cooling the lysate on ice for 30 min, it was centrifuged at 12000 × g for 10 min at 4°C. After denaturation, the proteins were separated by 12% SDS-PAGE, wet transferred onto a polyvinylidene fluoride membrane, blocked with blocking buffer (Beyotime, China), and rotated for 1 h at room temperature. The blots were incubated with monoclonal anti-human HIBADH (1:500; Abcam) or polyclonal β-actin (1:500; Beyotime, China) for 2 h at room temperature. The human HIBADH peptide sequences showed 96.13% homology with the corresponding bovine HIBADH protein. After washing the membranes thrice with 0.1% Tween-20 in 1× TBS for 5 min each time, goat anti-rabbit secondary antibodies (1:500; Beyotime, China) were incubated with the membranes to detect antigen-antibody complexes. The bound secondary antibodies were visualized using the 3,3′-diaminobenzidine tetrachloride (DAB; Cwbiochem, China) horseradish peroxidase color development kit (Beyotime, China) according to the instructions of the manufacturer. The molecular mass of the proteins were measured using a pre-stained protein ladder (Thermo, USA).

The testicular and epididymal tissues from two adult bulls were fixed in 4% paraformaldehyde. All tissues were embedded in paraffin and sectioned for IHC [14]. Deionized water (1 L) and 10 mL citrate buffer solution were used to rehabilitate the antigen. Subsequently, the antigen was washed with 0.01 M phosphate-buffered saline (PBS; pH 7.4) twice every 3 min. The immunoreaction slides were deparaffinized and hydrated. The slides were blocked with endogenous peroxidase for 10 min, washed with PBS, and incubated with the monoclonal anti-human HIBADH antibody (1:100; Abcam, HK, China) for 60 min at room temperature. After washing with PBS, the slides were incubated with goat anti-rabbit secondary antibody (1:100; Beyotime, China) for 15 min at room temperature. The antibodies were visualized with 0.6 mg/mL DAB horseradish peroxidase color development kit (Cwbiochem, China) for brown staining under a microscope (OLYMPUS BX53) according to the instructions of the manufacturer. The slides were stained with hematoxylin (Cwbiochem, China), dried, and photographed using a digital camera.

For the IFA, the sperm cells were mounted on slides, fixed in 4% paraformaldehyde, and washed with PBS twice every 5 min. The slides were sealed with 3% bovine serum albumin for 30 min at room temperature and incubated with monoclonal anti-human HIBADH antibody (1:150; Abcam, HK, China) overnight at 4°C. After washing with PBS, the slides were incubated with goat anti-rabbit secondary antibodies (1:150; Beyotime, China) for 60 min at room temperature and re-washed with PBS. The nucleus was stained with 4',6-diamidino-2-phenylindole. Finally, the cells were photographed using an inverted microscope (OLYMPUS).

### Genetic variations screening of 5′-flanking region in the *HIBADH* gene

Three primer pairs (Q-1, Q-2, and Q-3 F/R, Table 2) were designed using the primer PREMIER 5.0 to amplify the 5′-flanking region in the *HIBADH* gene based on the GenBank reference sequence of bovine *HIBADH* (Accession No. AC_000161.1). The primer pairs were synthesized by Shanghai Sangon Biological Engineering Co., Ltd. The PCR amplification fragments from 50 randomly selected samples were directly sequenced in both directions using an ABI 3730xl sequencing platform (Applied Biosystems, USA) by following the standard protocol. The sequencing results were analyzed using the DNASTAR 8.0 package (DNASTAR, Inc., USA) to detect genetic variations in the *HIBADH* gene.

**Table 2:** Primer information used for cloning the bovine *HIBADH* gene promoter region.

Primer names	Sequences (5′-3′)	Annealing temperature (℃)	The size of production (bp)
Q-1	F:GCTACCATGCCAAGAACCCT	62.5	1430
	R:TGCTAGGAACTTGCTCCTCAA		
Q-2	F:ACACACAAACATACTTTCTGCACT	59.7	1675
	R: GGCCCTCCAATTGTCCTCAA		
Q-3	F:TGTTTTGGGTGTCCATGGCT	61.1	1812
	R:GGCCCTCCAATTGTCCTCAA		
P-1	F:**CGGGGTACC**GTCAAAGAAAGGAACTGCT	61.9	2340
	R:**CCGCTCGAG**AATGATACGGAAGGTGGAA		
P-2	F:**CGGGGTACC**AGTCAGCTTCTCGAACCAGA	61.6	1805
	R:**CCGCTCGAG**AATGATACGGAAGGTGGAA		
P-3	F:**CGGGGTACC**AGAATCTATTCGGCAAATTA	63.2	1495
	R:**CCGCTCGAG**AATGATACGGAAGGTGGAA		
P-4	F:**CGGGGTACC**AGGCGCTGCGATTAGA	60.1	618
	R:**CCGCTCGAG**AATGATACGGAAGGTGGAA		
G	F:CTCCAACATCACAGTTCAAAAG	62.5	967
	R:TACCCGCTGCAAGGCT		
M-outer	F:ATTTGGGATTTTTGATTGATTTTT	52.2	218
	R:CTCACCCACTAACTAACAAAATAC		
M-inner	F:GGGGTTTGTAAGGTGAT	52.2	175
	R:GGCGGTAACAAAATACGTAATA		
PT	F:GCATGGCAGCCTCCTTACG	61.2	1254
	R:AGAGACCATCAGTGGCTTGC		

Note: The *Kpn* I sites (**CGGGGTACC**) were added to the forward primers (P-1, P-2, P-3, and P-4) as recognition and protection sites. The reverse primers (P-1, P-2, P-3, and P-4) (3′) contained the *Xho* I sites (**CCGCTCGAG**).

### Prediction of the core promoter region and the functional elements of the 5′-flanking region

The core promoter of bovine *HIBADH* was predicted using the Genomatix Software (http://www.genomatix.de/applications/index.html). The position of the TATA box was predicted using PROSCAN, version 1.7 (http://www-bimas.cit.nih.gov/cgi-bin/molbio/proscan). The transcription factors were predicted using TFSEARCH (version 1.3) (http://www.cbrc.jp/research/db/ TFSEARCH. html) and WWW Promoter Scan (http://www-bimas.cit.nih.gov/molbio/prosan/). The CpG islands were analyzed using the online software CpG Island Searcher (http://www.uscnorris.com/cpgislands2/cpg.html). The nucleic acid sequences were analyzed using accepted software formats.

### Genotyping and statistical analysis

DNA sequencing revealed that only one genetic variation (g.-165 T>C, rs133569227) was found in the 5′-flanking region of the bovine *HIBADH* gene. The gene was subsequently genotyped by PCR-restriction fragment length polymorphism (RFLP) analysis. The primer (G, F/R) designed for genotyping is shown in Table 2. The corresponding restriction endonuclease *Sma* I was selected for the digestion of the PCR products according to the recommendations of the manufacturer. The digested fragments were separated on 1% agarose gel. Electrophoresis was performed in 1× Tris-acetate EDTA buffer at a constant voltage of 110 V for 30 min at room temperature. Genomic DNA samples from a total of 307 Chinese Holstein bulls were genotyped.

The genotypic and allelic frequencies, Hardy–Weinberg equilibrium χ^2^ test, polymorphism information contents (*PIC*), heterozygosities (*H*_*e*_), and the effective population of the allele (*N*_*e*_) were calculated using Tools for Population Genetic Analyses software (http://www.marksgeneticsoftware.net/tfpga.htm), as described by Miller (1997) [36]. The associations between the SNP marker and sperm quality traits were analyzed after performing the general least-square model procedure from the Statistical Analysis Software (SAS Institute Inc., Cary, NC, USA, 2002). The linear model is represented as follows:

$$Y_{ijkl}=u+G_{i}+A_{k}+P_{j}+H_{l}+e_{ijkl}$$

where *Y*_*ijkl*_ is the observed value for each semen quality trait; *u* is the overall mean; *G*_*i*_ is the fixed effect of genotype; *A*_*k*_ is the fixed effect of age [*k* = 2 ∼ 10, (1) 2 years old to 3 years old; (2) 4 years old to 5 years old; and (3) 6 years old to 15 years old]; *P*_*j*_ is the fixed effect of the origin of the bull; *H*_*l*_ is the effect of the farm; and *e*_*ijkl*_ is the random residual error. The values with *P* < 0.05 were considered significant. The data are presented as mean ± standard error. Multiple comparisons were performed using the Duncan method [37].

### Sodium bisulfite treatment

The genomic DNA from the fresh semen samples was treated using a BisulFlash DNA modification kit (Epigentek, USA) according to the protocol of the manufacturer. The modified DNA was maintained at -20°C until PCR amplification.

### Nest PCR amplification, cloning, and bisulfite sequencing

Two primer pairs (M-*HIBADH-*outer F/R and M-*HIBADH-*inner F/R, Table 1) were designed for the nested PCR amplification using the MethPrimer program (http://www.urogene.org/cgi-bin/methprimer/methprimer.cgi). PCR was conducted at a final volume of 10 μL. The nested PCR conditions were as follows: the reaction system for the first round amplification consisted of 5 μL 2× GC buffer I (5 mM Mg^2+^ Plus), 1.7 μL 2.5 mM dNTP mixture, 0.1 μL TaKaRa LATaq (5 U/μL), 0.5 μL each of 10 μM M-*HIBADH*-out primers, and 1.5 μL template DNA; and for the second round of amplification, 0.5 μL template DNA (from the first round product) and M-*HIBADH*-inter primers were used. The PCR program proceeded as follows: initial denaturation at 94°C for 5 min; 45 cycles of 94°C for 40 s, 50.5°C annealing for 30 s, and 72°C for 1 min; and final extension at 72°C for 5 min. The PCR products were separated and detected on 2% agarose gel, purified using a Gel/PCR extraction kit (BioTeke, Beijing, China), cloned into the pEASY-T3 Vector (TransGen, China), and transformed into *E*. *coli* DH*5α* cells for clone sequencing. Only the sequences derived from the clones with >95% cytosine conversion were analyzed. The percentage of DNA methylation was calculated by counting the number of methylated CpGs from the total number of CpG sites in individual clones. The bovine *HIBADH* sequence (GenBank, Accession No. AC_000161.1) was used as a reference for methylation status analysis using the BiQ Analyzer software.

### Cloning and construction of *HIBADH* promoter-reporter plasmids

To evaluate the promoter activity of the different parts of the 5′-flanking region of the *HIBADH* gene, we made serial truncations of the *HIBADH* promoter fragment in the range -1548 bp to +792 bp, as follows: pGL3-1548, pGL3-1013, pGL3-703, and pGL3+175. We subsequently analyzed the activity of the reporter constructs. To obtain the core promoter region, the reporter construct pGL3-703→+175 (-703 bp to +175) was generated. The four pairs of primers (P-1, P-2, P-3, and P-4 F/R), which were progressively located closer to the transcription starting site of the *HIBADH* gene, are listed in Table 2. The forward and reverse primers contained restriction sites for *Kpn*I and XhoI, respectively. The *Kpn*I sites (GGGGTACC) were added to the primers as recognition and protection sites. The reverse primer (in the 3′ direction) contained an *Xho*I site (CCGCTCGAG). The amplified promoter fragments were purified, double-digested with the restriction enzymes, and cloned into the pGL3-basic luciferase reporter vector (Promega, Beijing, China). The recombinant plasmids were confirmed by sequencing.

To examine the effect of the SNP g.-165 T>C on *HIBADH* promoter activity, two reporter plasmids with T or C (pGL3-T and pGL3-C), which encompassed the core promoter region, were constructed.

### Transient transfection and luciferase reporter assays

MLTC-1 cells were obtained from the Cell Culture Collection of the Chinese Academy of Sciences, Shanghai, China. The MLTC-1 cell line was cultured in RPMI-1640 medium (Sigma Co., St. Louis, MO, USA) with 10% fetal bovine serum (Invitrogen Life Technologies) containing 10 mg/L of penicillin and streptomycin (Invitrogen Life Technologies) at 37 xC in 5% CO_2_. For the luciferase reporter assays, MLTC-1 cells were inoculated in 48-well plates and were grown to 70% to 80% confluence. Transfection was performed using the Lipofectamine 2000 reagent (Invitrogen), according to the instructions of the manufacturer. The cells were co-transfected with 50 ng pRL-TK vector DNA (Promega) and 400 ng of either the empty pGL3-Basic plasmid (a promoterless control from Promega) or one of the promoter constructs with different lengths and genotypes of the *HIBADH* promoter. The pRL-TK vector, which provided the constitutive expression of *Renilla* luciferase, was co-transfected as an internal control to correct differences in transfection and harvesting efficiency. After 48 h of incubation, the cells were washed, harvested, and analyzed for luciferase activity using the Dual-Luciferase Reporter Assay System (Promega). Promoter activity was reported in relative light units and normalized against the activity of the empty pGL3-basic vector. All transfections were performed in triplicate and repeated at least six times in independent experiments.

### Statistical analysis

The data are presented as means ± SE. The statistical significance for gene expression and promoter activity was tested using the Student t-test. The methylation patterns of the CpG sites of the *HIBADH* promoter were analyzed using Chi-square test. The differences between the groups were considered significant at *P < 0*.*05*.

## Results

### Expression and localization of bovine HIBADH Protein

**Fig 1:**
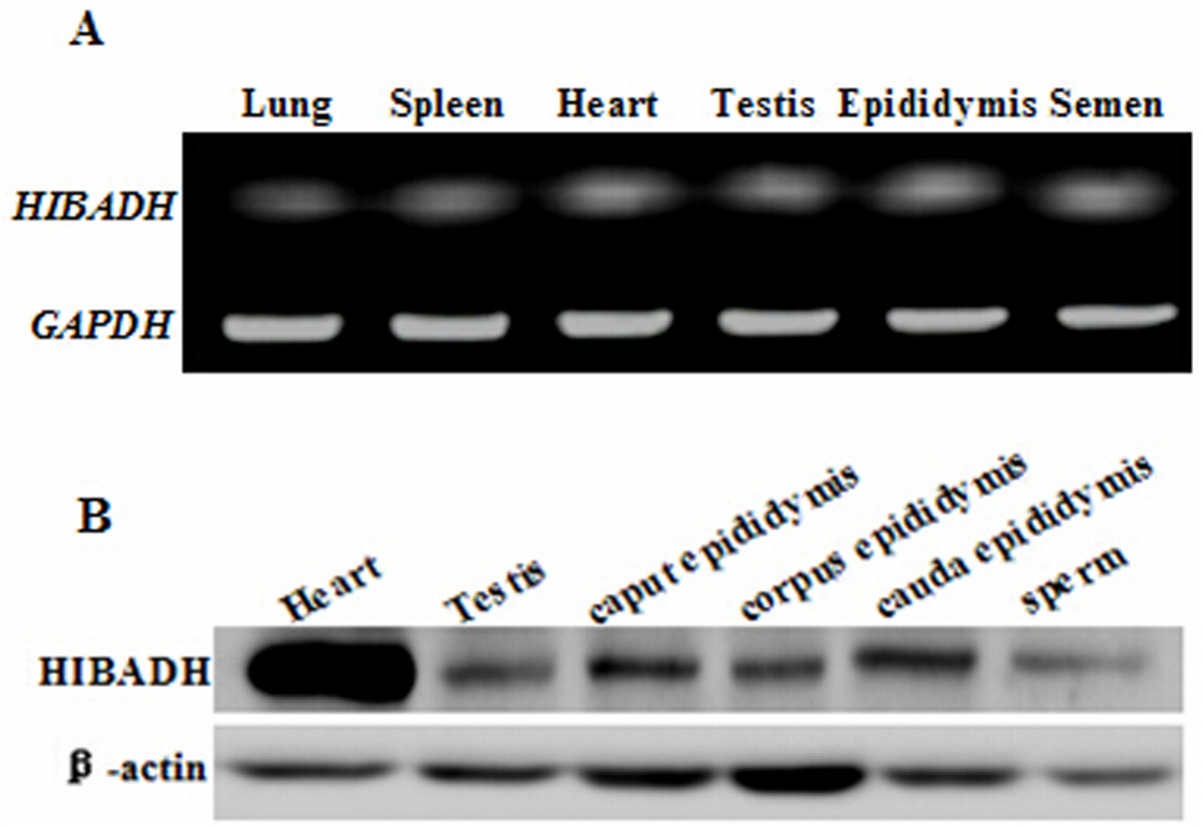
*HIBADH* expressed in different bull tissues. (A) The transcripts of *HIBADH* were detected by RT-PCR in lung, spleen, heart, testis, epididymis, and ejaculate semen. *GAPDH* was used as the positive control. (B) Western blot analysis of HIBADH in adult bull tissues and ejaculate (with β-actin as the control).

**Fig 2:**
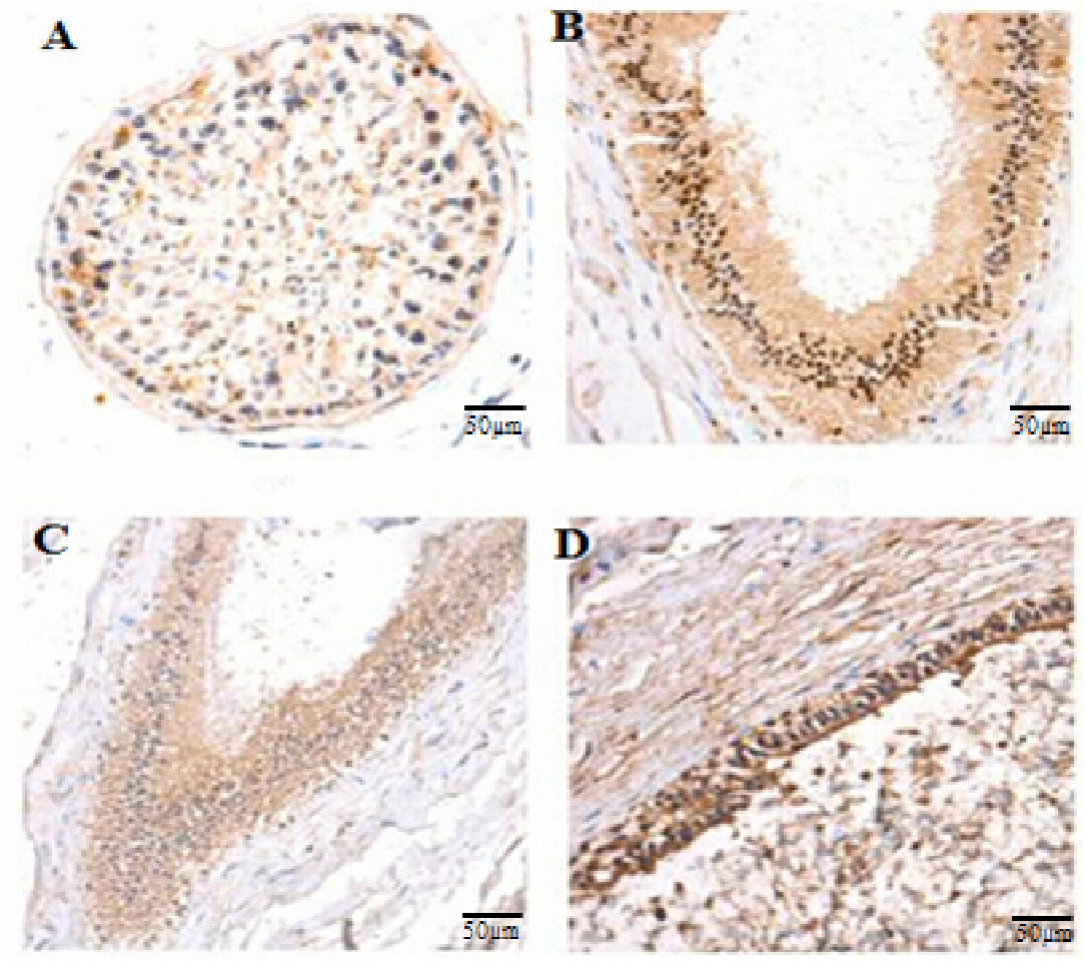
Immunolocalization of HIBADH in bull seminiferous epithelium and epididymis. (A, B, C, and D) Localization of HIBADH in adult bull testis, caput epididymis, corpus epididymis, and cauda epididymis, respectively. The brown area indicates the expressed protein, whereas the blue area is the nuclear DNA signal (negative control). These cells were photographed using an inverted microscope (OLYMPUS) at 40 × 10. Images were obtained on a 50 μm scale plate.

**Fig 3:**
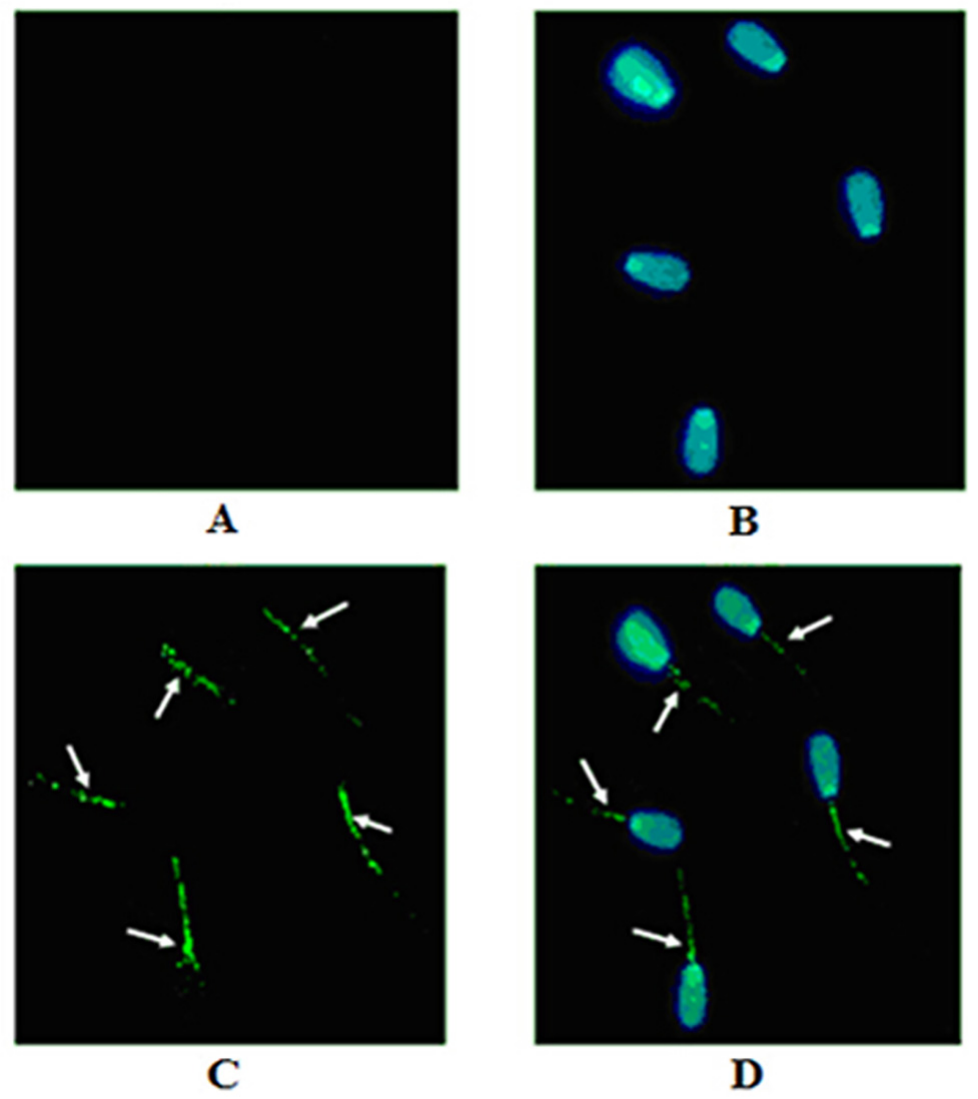
HIBADH is expressed and localized in bull spermatozoa. The arrow heads indicate that HIBADH protein was mainly localized at sperm neck-piece and mid-piece, and to a lesser extent, in the sperm head. (A) Negative control. (B) Nuclear DNA signals (blue). (C) HIBADH protein signals (green). (D) Merging of HIBADH protein (green) and nuclear DNA signals (blue). Image was obtained using an inverted microscope (OLYMPUS) at 40 × 10. Images were obtained on a 5 μm scale plate.

To validate the expression pattern of *HIBADH*, the bull tissue and semen samples were subjected to RT-PCR and Western blot analysis. The transcripts of *HIBADH* were present in the heart, spleen, lung, testis, epididymis, and semen of bull, as detected by RT-PCR (Fig 1A). Using Western blot analysis with anti-human HIBADH antibody, the HIBADH protein was expressed in bull heart, testis, caput epididymis, corpus epididymis, cauda epididymis, and sperm, and relatively higher signals were detected in the heart than in the other tissues (Fig 1B). To further understand HIBADH protein expression pattern during spermatogenesis, the testicular and epididymal morphologies of the bulls were analyzed by IHC. The HIBADH protein immunoreactivity was detected in the seminiferous epithelium (which included pachytene spermatocytes, primary spermatocytes, and spermatids), as shown in Fig 2. IHC revealed the HIBADH protein expression in epithelial cells throughout the entire bull epididymis, including caput epididymis, corpus epididymis, and cauda epididymis. By conducting thorough research by using IFA, we found that HIBADH was expressed in the neck and in the mid-piece, both of which contained the mitochondria in bull spermatids (Fig 3). HIBADH may play a role in maintaining sperm motility in Chinese Holstein bulls.

### Identification of genetic variants within the 5′-flanking region of *HIBADH* gene

We sequenced a 2,340 bp segment from the 5′-flanking region of the *HIBADH* gene in Chinese Holstein bulls. One reported SNP (g.-165 T>C, rs133569227) was discovered using sequence alignment and was compared with the *HIBADH* sequence (GenBank accession No. AC_000161.1) (Fig 1). Bioinformatics prediction showed that this SNP was located in the core region of the promoter.

### Genotyping the SNP by PCR-RFLP

**Fig 4:**
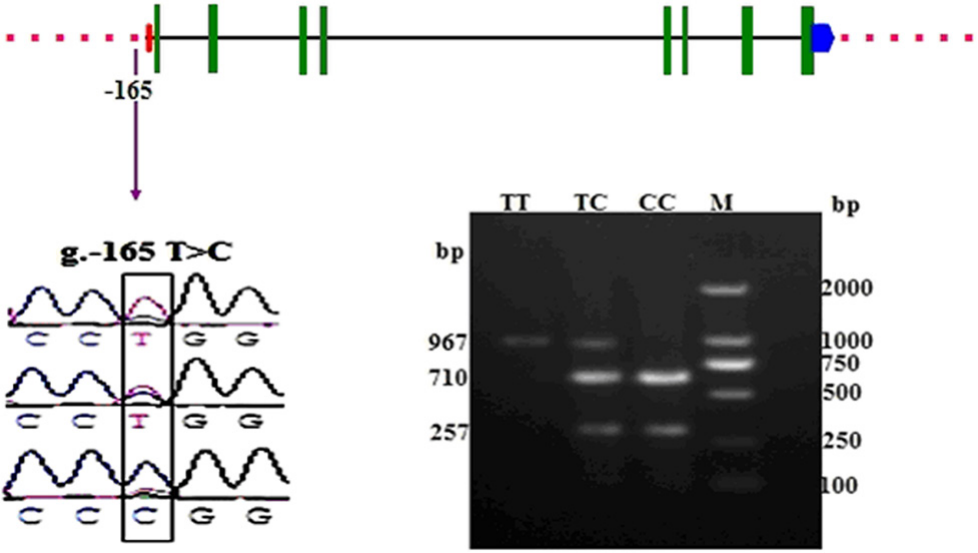
Structure of *HIBADH*, location of the identified SNP (g.-165 T>C), and PCR-RFLP patterns. Patterns for g.-165 T>C genotypes TT, TC, and CC. M: Marker. Digestion with *Sma* I of the amplified *HIBADH* gene g.-165 T>C locus produced fragments of the following sizes: 967 bp for genotype TT; 967, 710, and 257 bp for genotype TC; and 710 and 257 bp for genotype CC.

The SNP g.-165 T>C in 307 Chinese Holstein bulls was genotyped via PCR-RFLP. Amplified *HIBADH* gene g.-165 T>C locus digestion with *Sma* I produced fragments of the following sizes: 967 bp for genotype TT; 967, 710, and 257 bp for genotype TC; and 710 and 257 bp for genotype CC (Fig 4). The obtained genotypes were in agreement with the DNA sequencing results.

### Prediction of the promoter region of *HIBADH* gene

**Fig 5:**
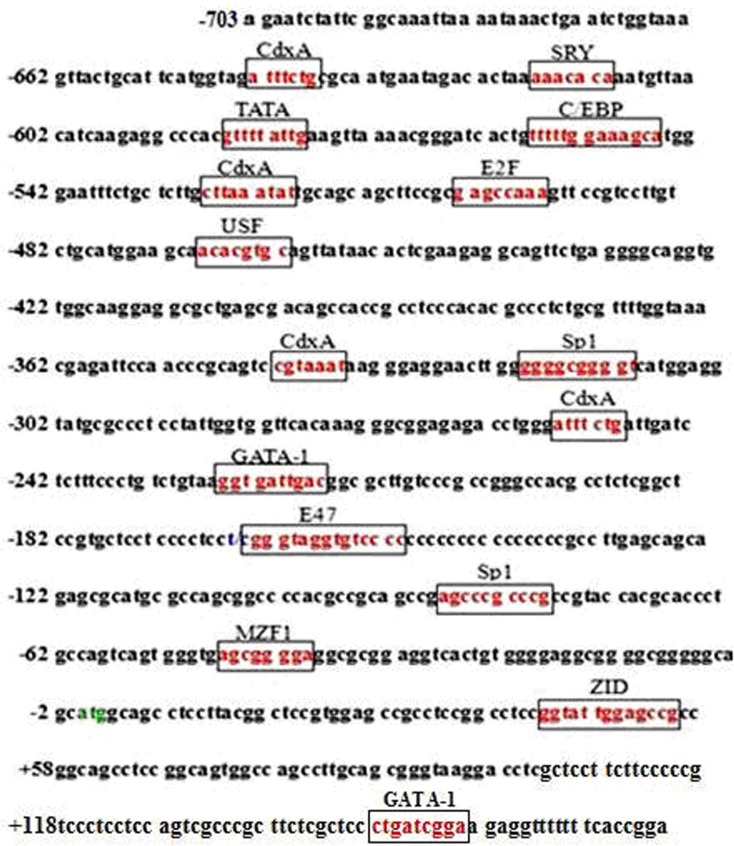
The core promoter region sequence of *HIBADH* from the -703 bp to +175 bp of Chinese Holstein bulls. The green-highlighted TSS is marked as A with +1 (the nucleotide sequence numbered +1 is the first A of the TSS). The numbering of nucleotides is relative to the ATG. The boxed sequences represent the putative transcription factor binding sites. The nucleotides highlighted in blue represent the previously reported SNP (g.-165 T>C, rs 133569227).

Based on bioinformatics prediction, we found that the core promoter region of *HIBADH* was located in the -728 bp to +101 bp region. No typical TATA box was observed in the core promoter region. However, two atypical TATA boxes were present at -342 bp to -333 bp (CGTAAATAAG) and +64 bp to +73 bp (CCTCCGGCAG) from the transcription start site (TSS; the nucleotide sequence numbered +1 is the first A of the TSS) [16]. In addition, we found some transcription factor binding sites, including the CdxA, SRY, C/EBP, E2F, USF, Sp1, GATA-1, and ZID functional elements, in the putative core promoter region (Fig 5). The SNP g.-165 T>C was found in the transcription factor (E47) binding site of the promoter core region, which was eliminated by the presence of allele T (Fig 5).

### Genetic parameter analysis of the SNP g.-165 T>C

Table 3 shows the allele and genotype frequencies and other genetic indices of the SNP g.-165 T>C in the bovine *HIBADH* gene. The T allele was the dominant allele of g.-165 T>C. One possible reason for this phenomenon may be the long-term breeding of these populations for different purposes. The results of other genetic indices (*PIC*, *He*, *Ne*, and *χ* ^2^) showed that the locus g.-165 T>C possessed intermediate genetic diversity (0.25 < *PIC* < 0.50) and was unable to meet the Hardy-Weinberg equilibrium (*x* ^2^ = 34.747, *P* < 0.05). The selection pressure on this SNP locus was strong. The differences in the genotypic frequencies among the Holstein bulls may have resulted from long-term artificial selection because the tested Holstein bulls were mostly bred by implanting imported embryos from USA or Canada.

**Table 3:** Genotypic frequencies, allelic frequencies, and genetic diversity (*PIC*, *He*, *Ne*, and *x* ^*2*^) of the bull *HIBADH* gene at position g.-165 T>C.

Locus	Genotype	Number	Genotypic frequency	Allele	Allelic frequency	*PIC*	*He*	*Ne*	*x* ^*2*^ (*P* value)
g.-165T>C	TT	208	0.678	T	0.788	0.278	0.334	1.501	34.747 (3.75E-09
	TC	68	0.221	C	0.212				
	CC	31	0.101						

Note: *He* = heterozygosities; *Ne* = effectivity of alleles; *PIC* = polymorphism information content.

### Association between single variation and semen quality traits in Chinese Holstein bulls

The effects of the g.-165 T>C on semen quality traits (ejaculate volume, sperm density, initial sperm motility, post-thaw cryopreserved sperm motility, and deformity rate) of the Chinese Holstein bulls are summarized in Table 4. The SNP g.-165 T>C was associated with initial sperm motility (*P < 0*.*05*). The bulls with the TT genotype had higher initial sperm motility than those with the CC genotype (*P < 0*.*05*). Furthermore, we found an E47 transcription factor binding motif CANNTG [38,39], which was created in the presence of the C allele at g.-165 T>C, but disappeared in the presence of the T allele.

**Table 4:** Least square means and SE for semen quality traits of different genotypes in the *HIBADH* gene of 307 Chinese Holstein bulls.

Loci	Genotype /sample	Ejaculate Volume (mL)	Initial sperm Motility (%)	Sperm density(×10^8^/mL)	Frozen semen motility (%)	Deformity rate (%)
g.-165T>C	TT/208	6.28 ± 0.13	74.16 ± 0.63^a^	11.14 ± 0.29	42.14 ± 0.41	14.55 ± 0.30
	TC/68	5.99 ± 0.22	73.26 ± 1.12^ab^	10.94 ± 0.35	42.05 ± 0.74	15.01 ± 0.46
	CC/31	6.06 ± 0.32	71.91 ± 1.59^b^	10.35 ± 0.50	41.27 ± 1.06	14.50 ± 0.70

Note: Means in the same column with different lowercase superscripts (a and b) are significantly different at *P < 0*.*05*.

### Promoter activity analysis of the *HIBADH* 5′-flanking region

**Fig 6:**
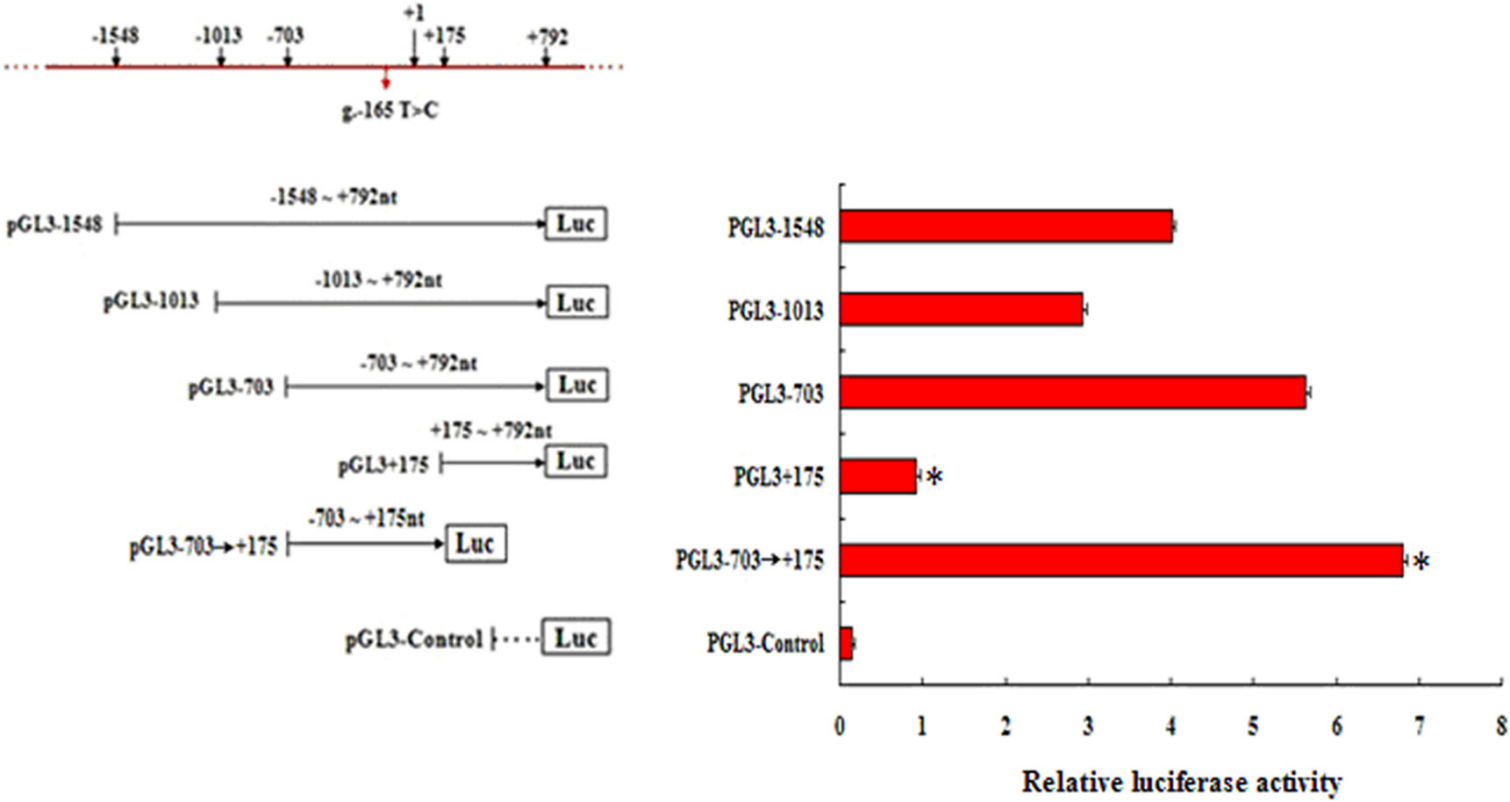
Scheme of the 5′-flanking region of the *HIBADH* gene and the identification of the core promoter region. The red arrow represents the previously reported SNP (g.-165 T>C, rs 133569227). Fragments pGL3-1548 (-1,548 bp to +792), pGL3-1013 (-1,013 bp to +792), pGL3-703 (-703 bp to +792), pGL3-703→+175 (-703 bp to +175), and pGL3+175 (+175 bp to +792) were amplified by PCR to produce the reporter constructs. Each fragment with wild-type alleles was cloned into the pGL3-Basic vector and transfected into MLTC-1 cells. Relative luciferase activity of a series of truncated constructs in the *HIBADH* 5x-flanking region was measured by dual luciferase assays of the MLTC-1 cells. For each construct, plasmid DNA extracted from 6 to 9 colonies was used. Results are presented as the average fold of firefly luciferase activity vs. the *Renilla* control vector (mean x S.D., n = 6 to 9). The asterisk indicates *P < 0*.*01* vs. the pGL3 basic control.

To determine whether the fragment -1,548 bp to +792 bp constituted an active promoter, we amplified a 2,340 bp fragment containing the 5′-flanking region of *HIBADH* (Fig 6). Subsequently, we generated truncated constructs (pGL3-1548, pGL3-1013, pGL3-703, and pGL3+175) by the progressive deletion of nucleotides from the 5′-end and cloned these fragments into the pGL3-Basic Luciferase vector (Fig 6). These constructs were transiently transfected into the MLTC-1 cells and were tested for luciferase activity to determine the shortest sequence required for the transcription of *HIBADH*.

As shown in Fig 6, the promoter activity of the basic pGL3 vector was significantly lower than that of all the constructs, and the pGL3-703→+175 (-703 bp to +175) had the highest promoter activity among all the constructs. The relative luciferase activity of the promoter pGL3-703 (1,459 bp) was upregulated by 3.442-fold compared with that of pGL3-1013 (1,805 bp) and by 3.602-fold compared with that of pGL3+175 (618 bp). Several negative regulatory elements may be present in the regions from -1,548 bp to -703 bp and from +175 bp to +792 bp. The results indicated that the *HIBADH* core promoter was located within the range of g.-703 bp to g.+175 bp.

**Fig 7:**
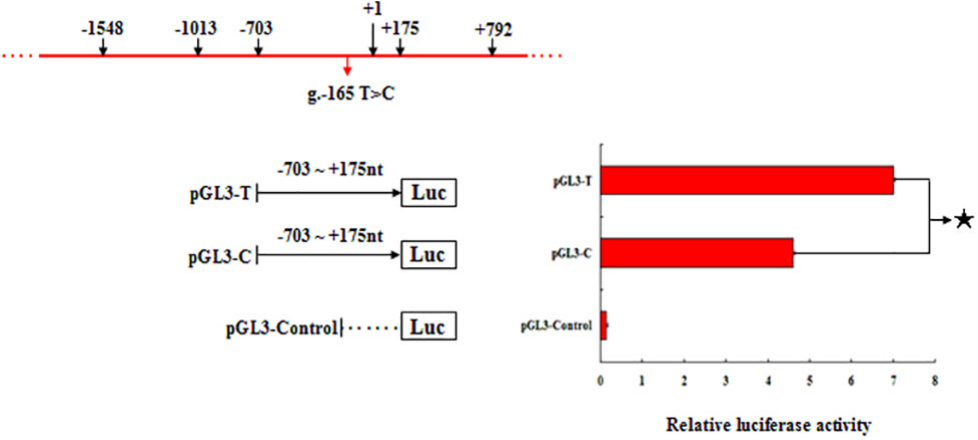
The effect of the SNP g.-165 T>C on *HIBADH* transcriptional activity in MLTC-1 cells. The 5′-flanking region of the *HIBADH* gene containing T or C loci (designated as pGL3-T and pGL3-C) from the *HIBADH* core promoter (-703 bp to +175 bp) was transiently transfected into MLTC-1 cells. The empty vector (pGL3) provided the lowest level of luciferase activity among all the loci constructs. The pGL3-T genotype showed 58% higher transcriptional activity than the genotype pGL3-C. For each construct, individual plasmid DNA extracted from 6 to 9 colonies was used. Results are presented as the average fold change of firefly luciferase activity vs. the *Renilla* control vector (mean ± S.D., n = 6 to 9) (Black star indicates *P < 0*.*05*).

The SNP g.-165 T>C was located in the core promoter region (g.-703 bp to g.+175 bp). To further investigate the effect of this SNP on *HIBADH* expression, the 5’-flanking region of the *HIBADH* gene, which contained the T or C loci (designated as pGL3-T and pGL3-C), was subjected to further functional analysis of the promoter activity. The different loci constructs from the *HIBADH* core promoter (-703 bp to +175 bp) were transiently transfected into MLTC-1 cells. As shown in Fig 7, the empty vector (pGL3) showed the lowest level of luciferase activity among all loci constructs. The pGL3-T genotype showed 58% higher transcriptional activity than the pGL3-C genotype. The SNP g.-165 T>C could be considered a functional variant and played a significant role in the transcriptional activity of *HIBADH* promoter.

### DNA methylation of the *HIBADH* promoter

**Fig 8:**
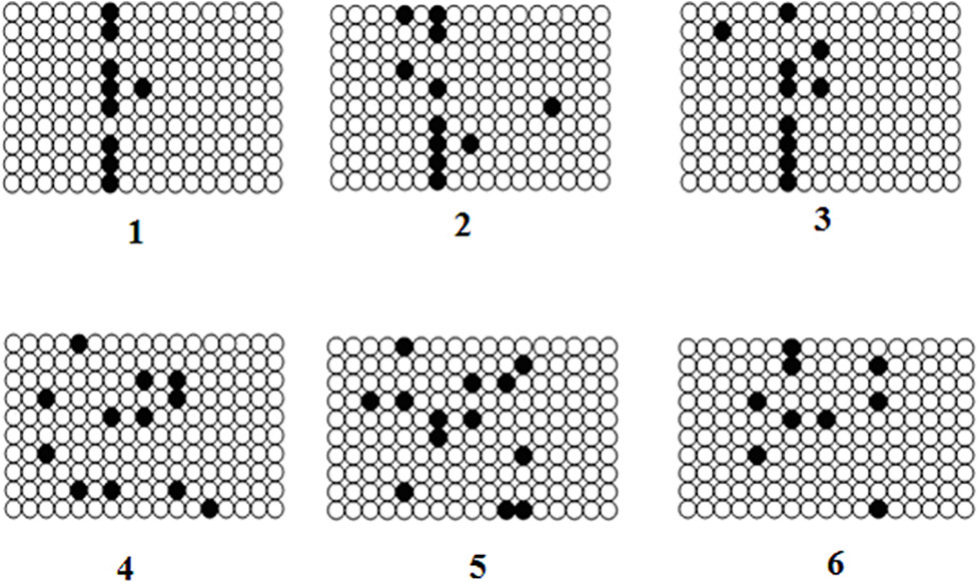
The promoter methylation profile of the HIBADH in the high-motility group (Bulls 1, 2, and 3) and the low-motility group (bulls 4, 5, and 6). We amplified a 175 bp fragment with 17 CpG sites in the core promoter region of each bull. Each fragment was cloned into a pEASY-T3 vector and transformed into *E*. *coli* DH5α cells for clone sequencing. Sixty selected clones (10 clones per bull) and a total of 1,020 CpG sites were analyzed. No significant difference in the frequency of DNA methylation of the *HIBADH* promoter between the high-performance (5.88%, n = 30 clones) and low-performance bulls (6.66%, n = 30 clones) (*P > 0*.*05*) was found, and all clones were hypomethylated (<50% of CpG sites on a given methylated strand). The 7^th^ CpG site showed higher methylation level in the high-motility group (73.33%, 22/30) than in the low-motility group (23.33%, 7/30) (*P < 0*.*0*1). Black circle represents Methylated CpG, Blank circle represents Unmethylated CpG.

To test whether the *HIBADH* promoter in bull was transcribed normally, the DNA methylation patterns of *HIBADH* in bull semen samples with different sperm motility values were detected using bisulfite sequencing. The bulls were divided into two groups, namely, a high-motility and a low-motility group (Table 5). We detected a CpG-rich domain in the core promoter using the online software CpG Island Searcher. Using the quantitative bisulfite sequencing method, we amplified the 175 bp fragment (-233 bp to -59 bp) with 17 CpG sites. Sixty selected clones (10 clones per bull) and a total of 1,020 CpGs were analyzed (Fig 8). We did not find any significant difference in the frequency of DNA methylation of the *HIBADH* promoter between the high-performing bulls (5.88%, n = 30 clones) and low-performing bulls (6.66%, n = 30 clones) (*P > 0*.*05*), and all clones were hypomethylated (< 50% of CpG sites on a given methylated strand) (Table 5, Fig 8). No difference was found in general, but a very specific methylation pattern was observed in the 7^th^ CpG site in the *HIBADH* core promoter. As shown in Table 5, the 7^th^ CpG site exhibited higher methylation level in the high-motility group (73.33%, 22/30) than in the low-motility group (23.33%, 7/30) (*P < 0*.*01*).

**Table 5:** Initial sperm motility parameters and promoter methylation levels of the six selected adult bulls.

Bull No.	Age (years)	Initial sperm motility (%)	Percentage of promoter methylation (%)	Percentage of methylation in the 7^th^ CpG site (%)
1	11	77.56	5.29	80.00
2	11	75.38	6.47	70.00
3	2.5	76.12	5.88	70.00
4	6	57.17	7.05	20.00
5	6	55.13	7.64	20.00
6	5.9	53.24	5.29	30.00

Note: Bulls 1, 2, and 3 belonged to the high-motility group, whereas bulls 4, 5, and 6 belonged to the low-motility group. We amplified a 175 bp fragment (-233 bp to -59 bp) with 17 CpG sites in the core promoter region of each bull. Each fragment was cloned into pEASY-T3 vector and transformed into *E*. *coli* DH5α cells for clone sequencing. Sixty selected clones (10 clones per bull) and 1,020 CpG sites were analyzed. No significant difference in the frequency of DNA methylation of the *HIBADH* promoter between the high-performing (5.88%, n = 30 clones) and low-performing bulls (6.66%, n = 30 clones) (*P > 0*.*05*) was found, and all clones were hypomethylated (<50% of CpG sites on a given methylated strand). The 7^th^ CpG site showed higher methylation level in the high-motility group (73.33%, 22/30) than in the low-motility group (23.33%, 7/30) (*P < 0*.*01*).

## Discussion

The bovine *HIBADH* is located on chromosome 4 and is composed of 8 exons (Fig 4) encoding a protein with 336 amino acids. HIBADH is highly conserved between different mammals, such as human, bovine, chimpanzee, monkey, dog, and mouse. For example, the bovine HIBADH peptide sequences showed 96.13% homology with the corresponding human HIBADH protein and 90.5% with murine HIBADH. Thus, the anti-human HIBADH mono-antibody was used for Western blot analysis, IHC, and IFA to detect the HIBADH expression pattern in bull in the absence of an anti-bovine HIBADH antibody. Previous studies revealed that *HIBADH* was expressed in rat liver, heart, muscle, kidney, and cultured neural cells [23,24], and *HIBADH* expression was high in the human cerebellum, heart, skeletal muscle, uterus, placenta, testes, and spermatozoa [25]. In the present study, we initially validated via RT-PCR that the transcripts of *HIBADH* were abundant in bull heart, spleen, lung, testis, epididymis, and semen. Furthermore, the HIBADH protein was expressed in bull heart, testis, caput epididymis, corpus epididymis, cauda epididymis, and sperm, and relatively higher signals were detected in the heart and cauda epididymis than in the other tissues. This kind of conservative and extensive expression may be associated with important functions because HIBADH, which is one of the critical enzymes that generate glucose in the gluconeogenesis pathway, is primarily expressed in the mitochondria of the liver, kidney, muscle, and cultured neural cells and may use valine, which is imported into the brain for energy generation [18, 20, 23]. The localization of the bovine HIBADH in the testes and epididymis suggests its role in spermatogenesis. We determined via IFA that the bovine HIBADH was located in the neck-piece and in the mid-piece of the sperm. In the sperm, mitochondria are located in the mid-piece and produced large quantities of adenosine triphosphate (ATP) by aerobic respiration. HIBADH is used to catalyze the oxidation of 3-hydroxyisobutyrate to methylmalonate semialdehyde in valine and is recognized as an ATP production-related protein in the mitochondria of spermatozoa. Its abundant presence in the mid-piece of the sperm suggested that the function of this protein may be associated with bovine sperm motility. Several studies also showed via the proteomic approach that HIBADH is a motility-related protein in humans [26, 27]. Previous reports showed evidence that this protein was highly expressed in the neck-piece and in the mid-piece of elongating, elongated, and mature sperms containing mitochondria during human spermiogenesis [25]. In Holstein-Friesian bulls, the potential role of HIBADH in sperm motility was confirmed via genome-wide association study [28].

The association analysis showed that the SNP g.-165 T>C was significantly correlated with the sperm quality traits tested, if we disregard the environment and peculiarities in the AI stations. This is the first report on the association between *HIBADH* gene polymorphisms and sperm motility traits in Chinese Holstein bulls. Sperm motility is a critical trait that allows sperm to move from the vagina into the cervix and to move again at the time of interaction with the egg (during fertilization). Thus, adequate sperm motility is vital for successful fertilization [40]. A number of possible interpretations support our findings. First, our results are similar to the analytical results in Holstein-Friesian bulls [28] and humans [25, 18]. HIBADH is involved in sperm motility via the mitochondrial function of spermatozoa. Hence, the variations in HIBADH may contribute to sperm quality traits. Second, the SNP g.-165 T>C may regulate the expression of bovine *HIBADH* gene via transcription factor E47 and may cause significant potential phenotype diversity. For the *HIBADH* SNP g.-165 T>C, the transcription factor E47, an alkaline (basic Helix-Loop-Helix) protein, could appear in the presence of the C allele, but could disappear in the presence of the T allele. As an important transcription factor of eukaryotes, the E47 factor could bind to their response element in the gene promoter to regulate the expression of different target genes and could be involved in the promotion of cell aging [38,39]. A previous study revealed that the potential phenotype diversity may be caused by the genetic variation at the transcription factor binding site [41]. In fact, the polymorphisms in the core promoter region could affect gene expression via transcription factors. For example, the promoter region polymorphisms in the human *MBL2* gene controlled the baseline expression of MBL2 [42]. The haplotypes HY, LY, and LX were correlated with high, intermediate, and low mannose-binding lectin levels, respectively [42,43]. A promoter SNP (g.-456 G>A) of the bovine phospholipase C zeta (*PLCz*) gene can regulate gene expression via a transcription factor (CAP/AP1) and can be associated with semen quality traits [16]. Third, we found that the bulls with genotype TT at g.-165 T>C showed relatively higher initial sperm motility than those with the CC genotype (*P < 0*.*05*). The core promoter (-703 bp to +175 bp) of the *HIBADH* gene containing the T loci pGL3-T genotype showed 58% higher transcriptional activity than the genotype pGL3-C, whereas the empty vector (pGL3) provided the lowest level of luciferase activity than the loci constructs. The different genotypes exhibited different promoter activities, thereby implying that genetic variation would likely regulate *HIBADH* expression. The bulls with the TT genotype may show higher *HIBADH* expression than those with the CC genotypes. Considering that the bulls with the TT genotype had significantly higher initial sperm motility than the bulls with the CC genotype, we suggested that the high expression of the bovine *HIBADH* gene was conducive to the maintenance of high sperm motility. Fourth, the bovine HIBADH was located in the neck-piece and in the mid-piece of the sperm, as shown in the IFA. Finally, the methylation of the 5′ promoters of DNA suppressed gene expression. DNA methylation defects in genes are reportedly associated with impaired human sperm production and quality [44,45]. In this study, we found no difference in the *HIBADH* promoter methylation between the groups with different sperm motilities, and all clones were in a state of hypomethylation. This demonstrated that the 5′ flanking promoter of *HIBADH* is hypomethylated in ejaculated semen, and its methylation pattern is unrelated to sperm motility. Thus, the expression of *HIBADH* played an important role in maintaining the motility of the sperm. In mammals, tissue-specific methylation and cell-specific methylation occur in a small percentage of 5′ CpG island promoters, whereas a much larger proportion occurs across gene bodies. Intragenic methylation is involved in regulating cell context-specific alternative promoters in gene bodies [46]. However, the 7^th^ CpG site showed abnormal methylation between different sperm motility groups. Whether these changes were accompanied by other disturbances and further affected sperm motility was unclear.

We were the first to report on the core promoter of the bovine *HIBADH* gene. The method of generating truncated constructs was used. The pGL3-1013 and pGL3+175 fragments had a significantly higher effect on the decreasing promoter activity. However, the fragment pGL3-703→+175 (-703 bp to +175) had the highest relative luciferase activity promoter activity compared with the other fragments (pGL3-1548, pGL3-1013, pGL3-703, pGL3+175, and pGL3-basis vector). Therefore, the *HIBADH* core promoter was limited to an 878 bp region (g.-703 bp to g.+175 bp), which was consistent with the predicted results of the bioinformatics analysis. The cause of the masking effect of these fragments was unclear. One possible reason was the regulation of transcription factors, including CdxA, E2F, GATA, and TATA box motif. These upstream regulatory elements could interact with each other and could be identified by extracellular or intracellular stimuli, thereby leading to the determination of the activity of the bovine *HIBADH* promoter. We chose MLTC-1 cell line for our experiments because of the high transfection efficiency in MLTC-1 and the high amino acid sequence homology (90.18%) between bovine and murine *HIBADH*.

In summary, functional SNP g.-165 T>C at the core promoter region rather than methylation played a role in the regulation of *HIBADH* expression in the testes and sperm. Our findings suggested that the bovine *HIBADH* gene may be a sperm motility-related gene.
